# Supplementary material for: A Multi-Elements Isotope Approach to Assess the Geographic Provenance of Manila Clams (Ruditapes philippinarum) via Recombining Appropriate Elements
Source: Foods. 2021 Mar 18;10(3):646. doi: 10.3390/foods10030646 (PMC8003290; doi:10.3390/foods10030646)
Supplement: Supplementary file 1 [file foods-10-00646-s001.pdf]

<Supplementary Materials>

A Multi-Elements Isotope Approach to Assess the Geographic Provenance of Manila Clams (*Ruditapes philippinarum*) via Recombining Appropriate Elements

Eun-Ji Won, Seung Hee Kim, Young–Shin Go, K. Suresh Kumar, Min-Seob Kim, Suk-Hee Yoon, Germain Bayon, Jung-Hyun Kim, Kyung-Hoon Shin

Supplementary Figures

Figure S1. Scatter plots for $\delta^{13}C$ and $\delta^{15}N$ values for clam samples collected from China, DPR Korea and Korea ······ 1

Figure S2. Grouping of Manila clams from three countries based on LDA analysis of $\delta^{13}C$, $\delta^{15}N$, $\delta^{18}O$, and δD. Bold italics indicate the specimens were not well identified by geographic origin ······ 2

Figure S3. Grouping of Manila clams from three countries based on LDA analysis of $\delta^{13}C$, $\delta^{15}N$, δD, and $\delta^{34}S$. Bold italics indicate the specimens were not well identified by geographic origin ······ 3

Figure S4. Grouping of Manila clams from three countries based on LDA analysis of $\delta^{13}C$, $\delta^{15}N$, $\delta^{18}O$, δD, and $\delta^{34}S$. Bold italics indicate the specimens were not well identified by geographic origin ······ 4

Figure S5. Grouping of Manila clams from three countries based on LDA analysis of $\delta^{13}C$, $\delta^{15}N$, δD, $\delta^{34}S$, and $^{87}Sr/^{86}Sr$. Bold italics indicate the specimens were not well identified by geographic origin ······ 5

Figure S6. Grouping of Manila clams from three countries based on LDA analysis of $\delta^{13}C$, $\delta^{15}N$, δD, $\delta^{18}O$, $\delta^{34}S$, and $^{87}Sr/^{86}Sr$. Bold italics indicate the specimens were not well identified by geographic origin ······ 6

Figure S7. Grouping of Manila clams from three countries based on LDA analysis of $\delta^{13}C$, $\delta^{15}N$, δD, $\delta^{18}O$, $\delta^{34}S$, and δNd. Bold italics indicate the specimens were not well identified by geographic origin ······ 7

Figure S8. Grouping of Manila clams from three countries based on LDA analysis of $\delta^{13}C$, $\delta^{15}N$, δD, $\delta^{18}O$, $\delta^{34}S$, δNd, and $^{87}Sr/^{86}Sr$. Bold italics indicate the specimens were not well identified by geographic origin ······ 8

*Corresponding author. E-mail: shinkh@hanyang.ac.kr; Tel.: +82-31-400-5536 (K.-H. Shin).

Supplementary Figures

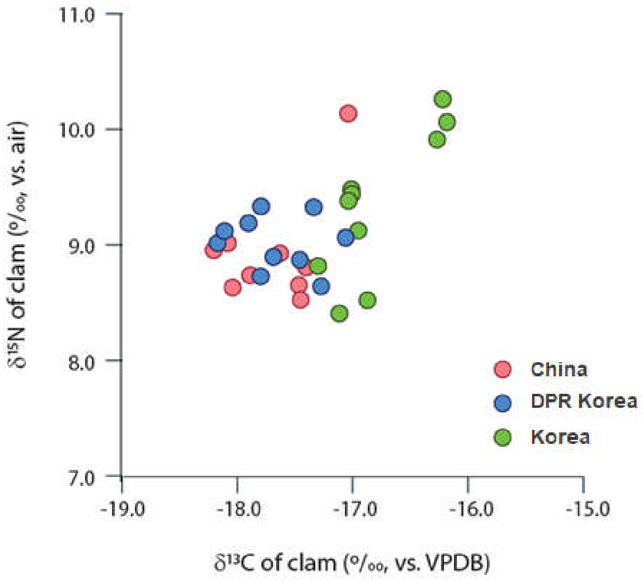

Figure S1. Scatter plots for $\delta^{13}C$ and $\delta^{15}N$ values for clam samples collected from China, DRP Korea and Korea

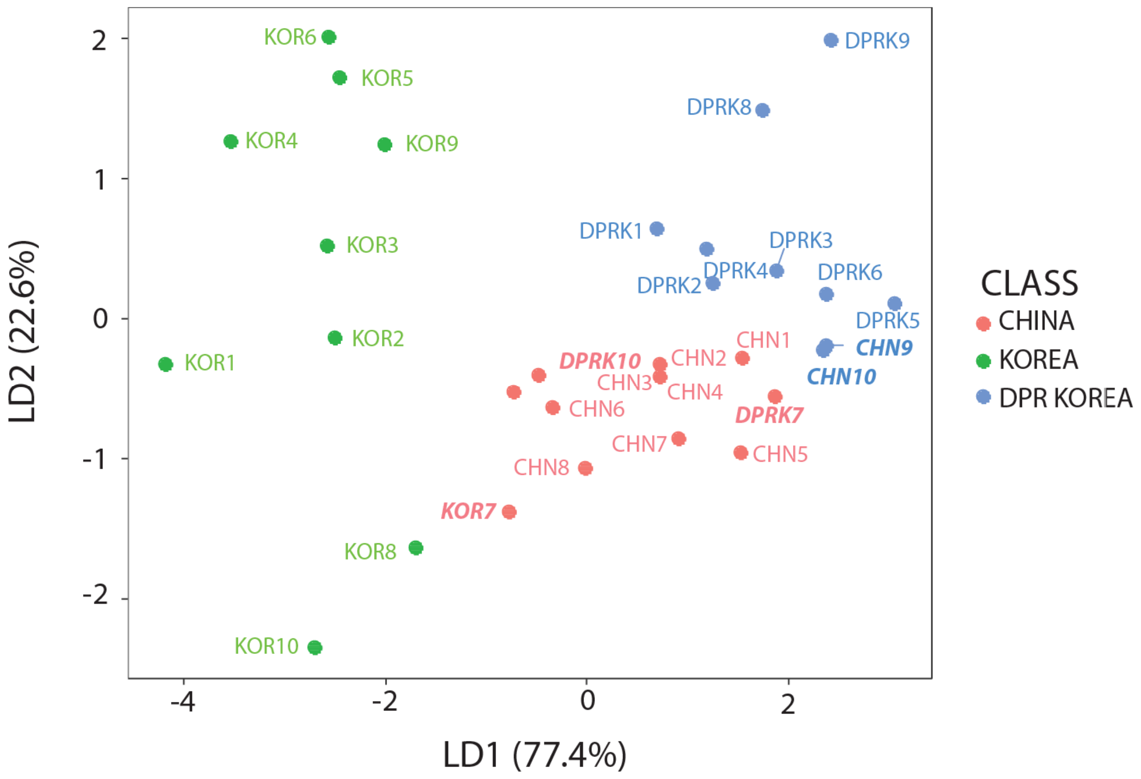

Figure S2. Grouping of Manila clams from three countries based on LDA analysis of $\delta^{13}C$, $\delta^{15}N$, $\delta^{18}O$, and δD. Bold italics indicate the specimens were not well identified by geographic origin

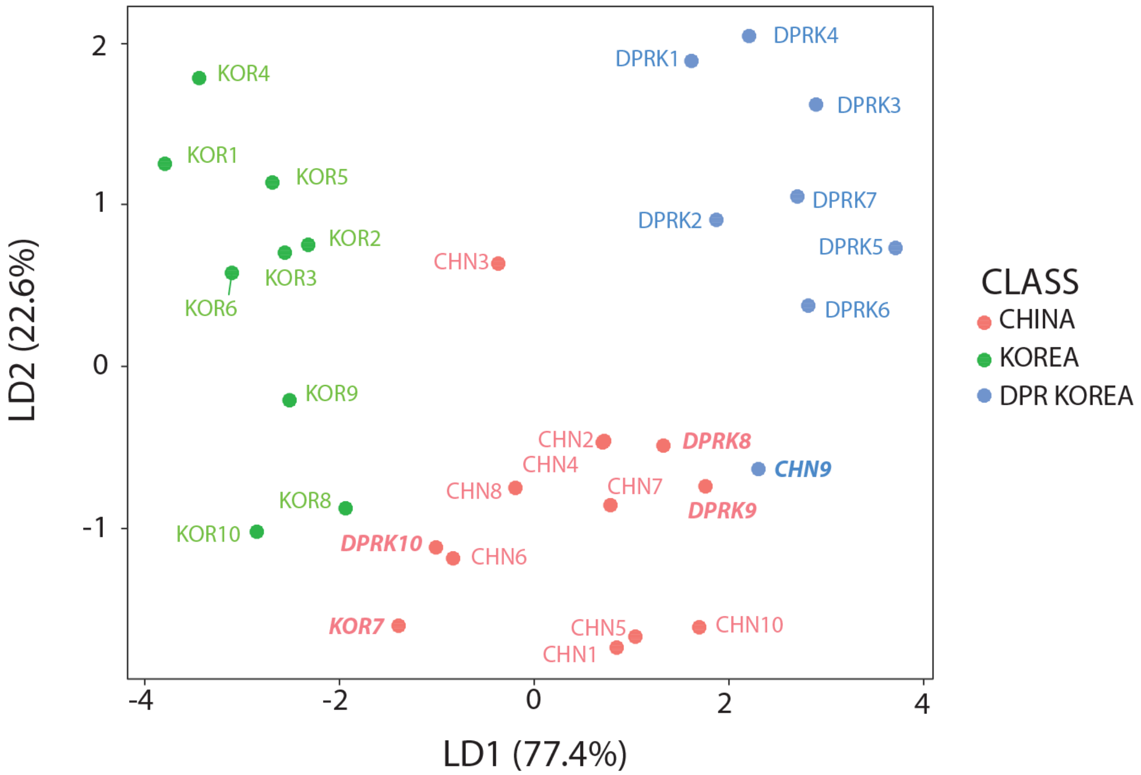

Figure S3. Grouping of Manila clams from three countries based on LDA analysis of $\delta^{13}C$, $\delta^{15}N$, δD, $\delta^{34}S$. Bold italics indicate the specimens were not well identified by geographic origin

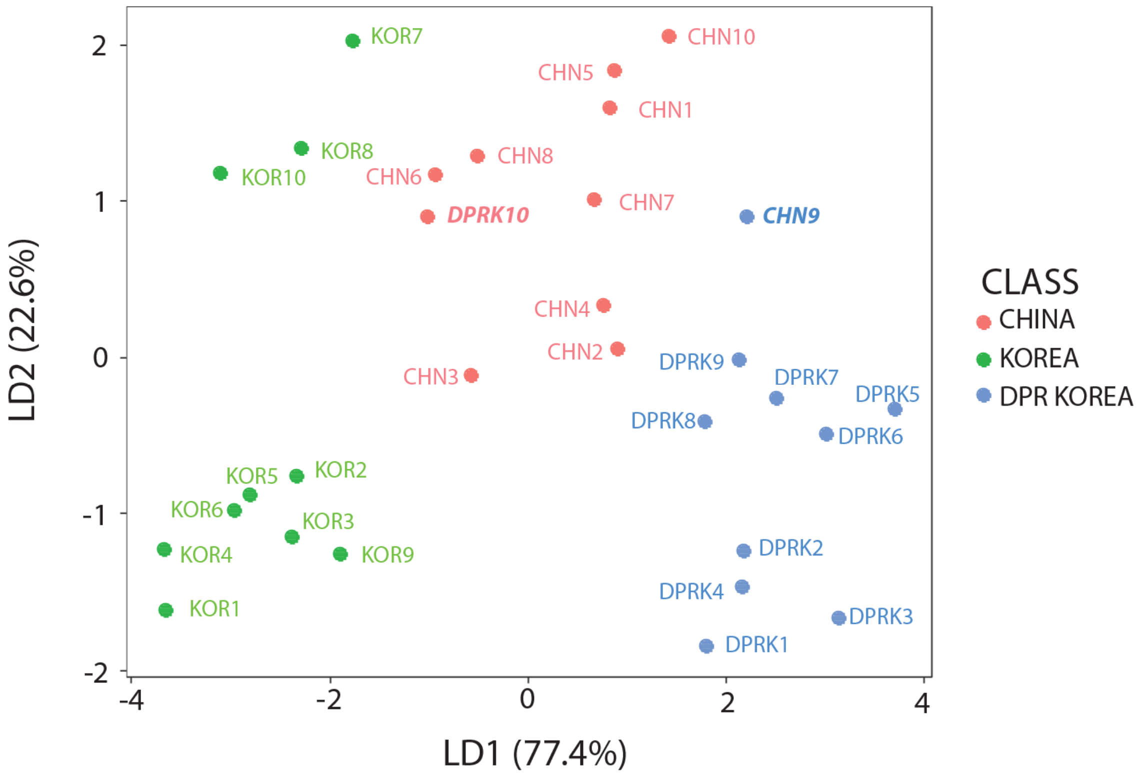

Figure S4. Grouping of Manila clams from three countries based on LDA analysis of $\delta^{13}C$, $\delta^{15}N$, $\delta^{18}O$, δD, and $\delta^{34}S$. Bold italics indicate the specimens were not well identified by geographic origin

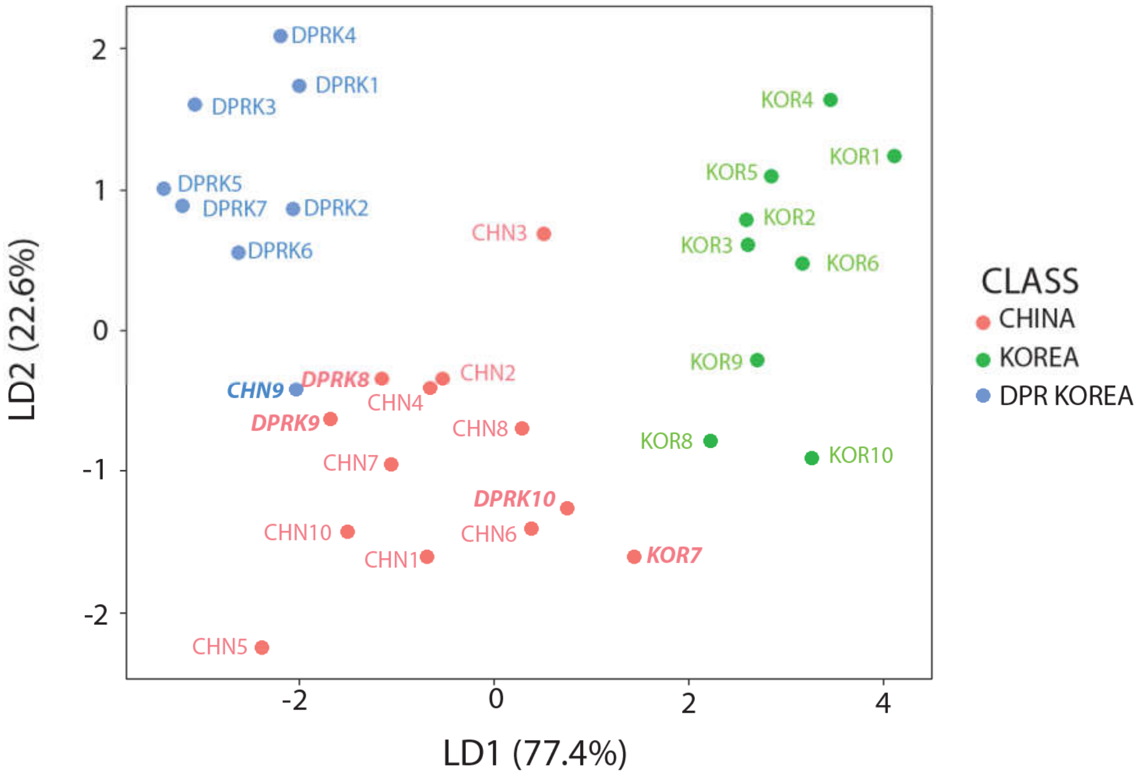

Figure S5. Grouping of Manila clams from three countries based on LDA analysis of $\delta^{13}C$, $\delta^{15}N$, δD, $\delta^{34}S$, and $^{87}Sr/^{86}Sr$. Bold italics indicate the specimens were not well identified by geographic origin

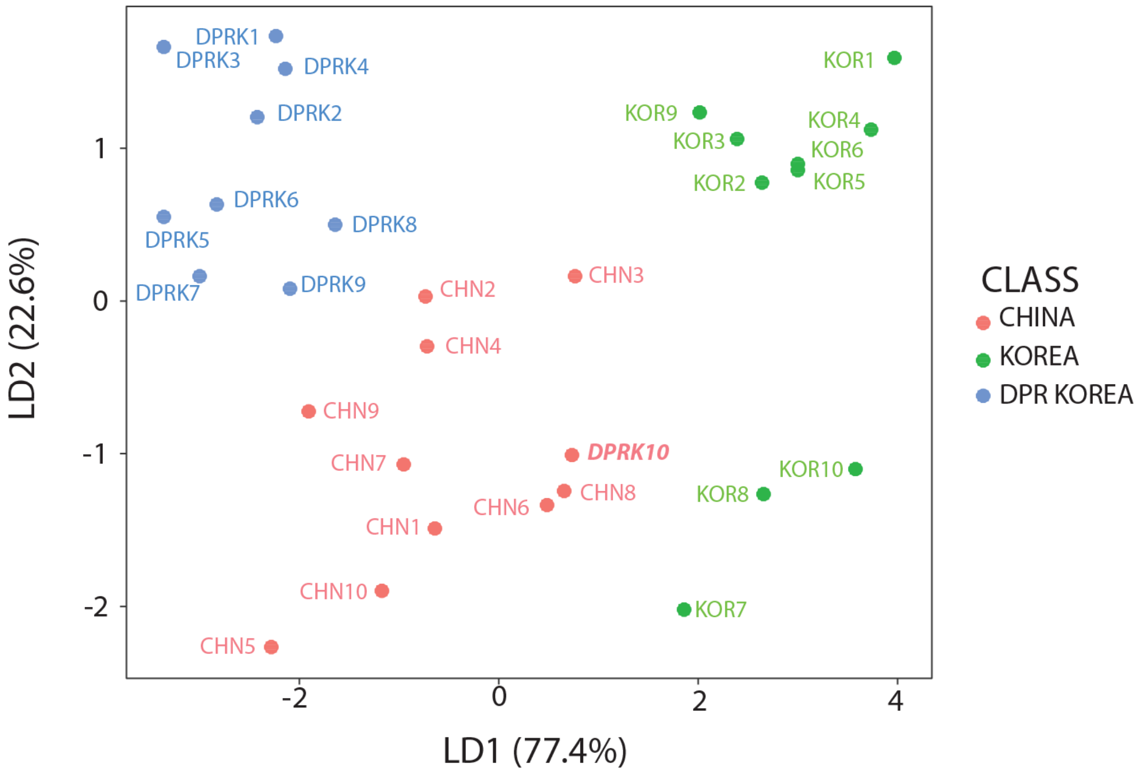

Figure S6. Grouping of Manila clams from three countries based on LDA analysis of $\delta^{13}C$, $\delta^{15}N$, δD, $\delta^{18}O$, $\delta^{34}S$, and $^{87}Sr/^{86}Sr$. Bold italics indicate the specimens were not well identified by geographic origin

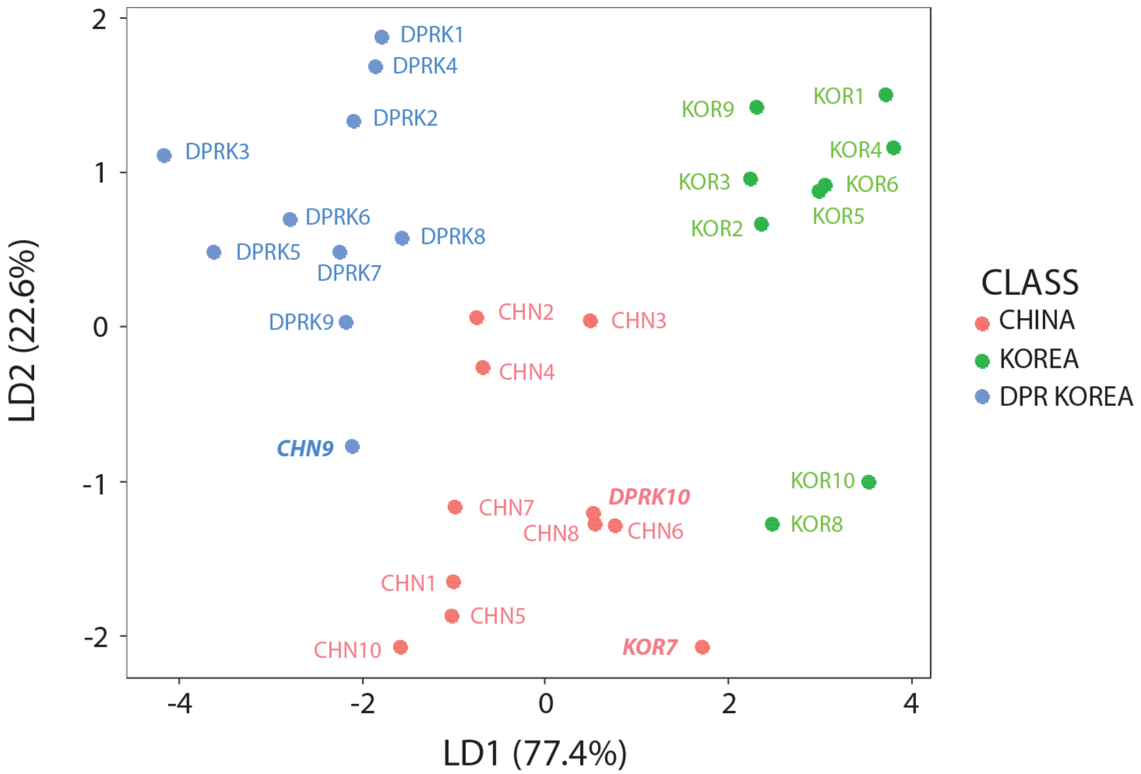

Figure S7. Grouping of Manila clams from three countries based on LDA analysis of $\delta^{13}C$, $\delta^{15}N$, δD, $\delta^{18}O$, $\delta^{34}S$, and εNd. Bold italics indicate the specimens were not well identified by geographic origin

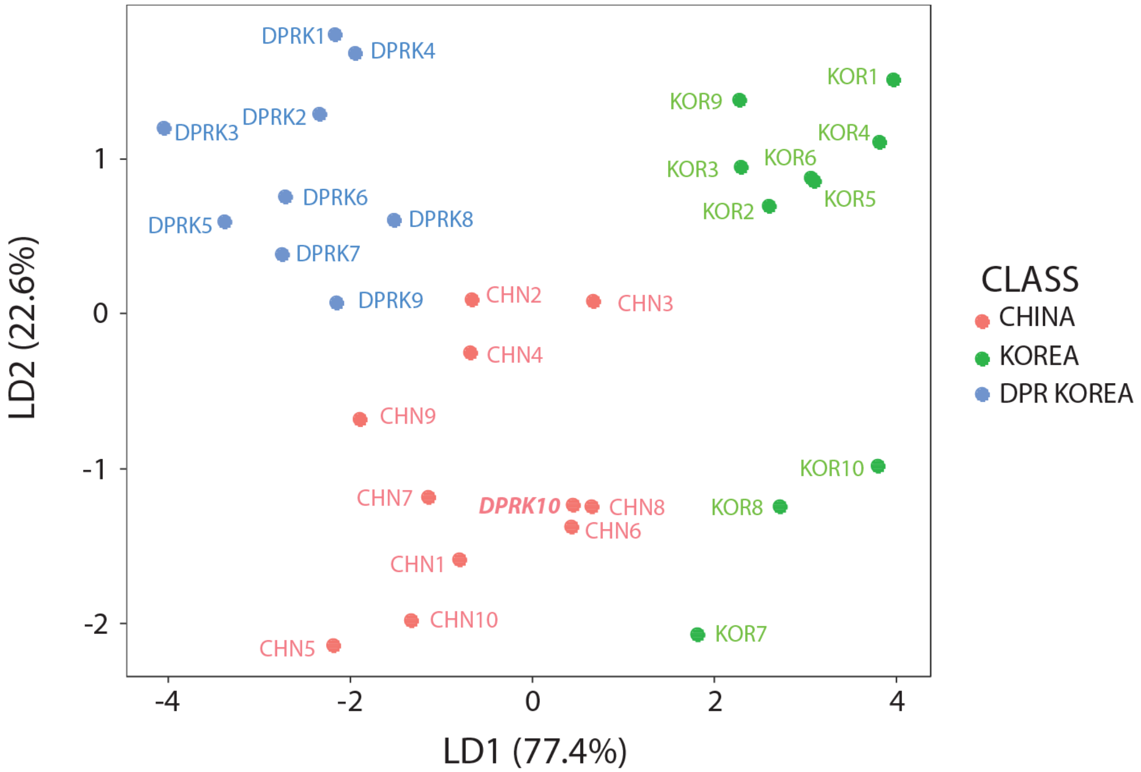

Figure S8. Grouping of Manila clams from three countries based on LDA analysis of $\delta^{13}C$, $\delta^{15}N$, δD, $\delta^{18}O$, $\delta^{34}S$, εNd, and $^{87}Sr/^{86}Sr$. Bold italics indicate the specimens were not well identified by geographic origin
